# A Century-Old Solution for 21st Century Challenges: Current Applications with a Focus on Biocontrol, Environmental Impacts, and Regulatory Perspectives

**DOI:** 10.3390/antibiotics15020180

**Published:** 2026-02-06

**Authors:** Anaelle Baud, Inès Rougis, Franck Bertolla

**Affiliations:** Laboratoire d’Ecologie Microbienne, UMR CNRS 5557, UMR INRAE 1418, VetAgro Sup, Universite Claude Bernard Lyon 1, 69622 Villeurbanne, France; ines.rougis@univ-lyon1.fr

**Keywords:** bacteriophage, One Health, phage therapy, phage-based plant protection, environmental safety, phage-bacteria coevolution, regulatory frameworks, agroecological transition

## Abstract

In the face of rising antimicrobial resistance, food insecurity, and climate change, bacteriophages are gaining renewed attention as promising biological alternatives to antibiotics across human, animal, and plant health sectors. Their high specificity, self-replicating capacity, and biodegradability make them valuable tools for combating antimicrobial or pesticide resistance and promoting sustainable biocontrol. This review discusses commonly accepted selection criteria for therapeutic phages, such as avoiding temperate types and screening for undesirable genes, while acknowledging ongoing debates and exceptions in specific clinical or ecological contexts. An overview of phage-based applications within a One Health framework is provided, spanning human medicine, veterinary practice, aquaculture, food safety and crop protection. Particular attention is given to agricultural biocontrol, where several successful plant protection strategies are highlighted, illustrating the feasibility and diversity of phage-based approaches. Despite their potential, key challenges remain regarding phage stability, formulation, and persistence under environmental conditions. Emerging innovations such as encapsulation, carrier bacteria, and protective formulations aim to enhance field performance. Furthermore, this review emphasizes the need to assess the environmental safety of phage applications, particularly their impacts on natural ecosystems, microbial communities, and ecological functions. Finally, the regulatory and policy challenges that currently limit the large-scale deployment of phages, particularly in the European Union, where they remain evaluated under conventional chemical pesticide frameworks are discussed. The development of dedicated regulatory pathways, better adapted to the specificities of phages, is argued to be essential for supporting their integration into agroecological transition strategies and next-generation antimicrobial policies.

## 1. Therapeutic Potential of Bacteriophages: Key Criteria and Safety Considerations

### 1.1. Fundamental Biological Characteristics of Bacteriophages

Shortly after their discovery in the early 20th century, bacteriophages attracted significant interest due to their therapeutic potential, particularly in human and veterinary medicine, as well as in the management of plant diseases. As early as the 1920s and 1930s, several studies reported their effectiveness in various infectious contexts. However, at that time, understanding of their biological properties and mechanisms of action remained very limited. The discovery of penicillin in 1928, followed by its rapid industrial-scale production, led to a decline in phage research and use, as antibiotics were considered easier to apply and offered broad-spectrum activity [1].

In recent years, however, the global crisis of antimicrobial resistance, along with concerns related to climate change and food security, has brought bacteriophages back into the spotlight. Phages exhibit several characteristics that make them highly attractive for therapeutic applications. Notably, they exert a highly specific antibacterial effect, with a host range often restricted to the strain level. This allows the targeted elimination of pathogenic bacteria without directly disrupting the surrounding microbiota [2]. Their safety for eukaryotic cells has been demonstrated in numerous studies [3]. In addition, phages are self-replicating in the presence of their bacterial host, a phenomenon referred to as auto-dosing: their numbers increase as long as the target bacterium is present and naturally decline in its absence [4]. Their biodegradability and limited persistence in treated environments make them particularly safe therapeutic agents [5].

However, recent findings suggest that some phages may exhibit broader host ranges than previously assumed, challenging the traditional paradigm of strict specificity [6]. This has sparked debate regarding the therapeutic relevance of polyvalent phages, which may offer broader protection but also raise questions about ecological safety and off-target effects.

### 1.2. Selection Criteria for Safe and Effective Use of Bacteriophages

Despite the lack of a unified regulatory framework, the scientific literature consistently highlights a number of minimum criteria required to ensure the safety and efficacy of bacteriophages used in human and veterinary therapeutics, as well as in biocontrol applications. These requirements are based on both phenotypic characterization and comprehensive genomic analysis of candidate phages.

The most consistently applied selection criterion is the exclusion of temperate phages in favor of strictly lytic ones. Indeed, the integration of viral DNA into the bacterial genome during the lysogenic cycle delays the immediate bactericidal effect, thereby compromising therapeutic efficacy. Moreover, lysogeny can provide the host bacterium with resistance to further phage infections through mechanisms including transcriptional repression of superinfecting phages (superinfection immunity; [7]) and inhibition of phage DNA entry (superinfection exclusion; [8]). In addition, prophages can induce lysogenic conversion, significantly altering the phenotype of the host bacterium. This phenomenon plays a critical role in the virulence of many pathogenic species. For example, the β-phage (a siphovirus) integrated into *Corynebacterium diphtheriae* encodes the diphtheria toxin Tox [9], and lambdoid phages are responsible for the expression of Shiga toxins in enterohemorrhagic *Escherichia coli* strains [10]. Temperate phages may also facilitate horizontal gene transfer through transduction, a process whereby bacterial DNA fragments are accidentally packaged and transferred to other cells. To minimize these risks, several bioinformatic tools have been developed to predict phage lifestyle, including Phage AI [11], PhageLeads [12], PHASTEST [13], and BACPHLIP [14], which rely on the detection of signature genes such as integrases, transposases, or plasmid replication origins. In addition, recent deep learning-based tools such as PhaTYP and DeePhage have been developed for lifestyle prediction, particularly in metagenomic contexts involving short contigs. However, their application in therapeutic workflows remains limited [15,16].

The systematic exclusion of temperate phages is currently a subject of debate [17]. When isolation of strictly lytic phages is particularly difficult for certain bacterial species, the use of temperate phages may be considered. The presence of at least one functional prophage in over 75% of the 13,700 bacterial genomes analyzed underscores their ubiquity and potential functional relevance [18]. In this context, both temperate prophages which can be genetically modified to inactivate lysogeny-related functions and lytic phage genomes unexpectedly detected within bacterial genome assemblies may serve as alternative reservoirs for therapeutic candidates. A recent large-scale bioinformatic analysis identified so-called bacterial assembly-associated phage sequences, including complete lytic phage genomes closely related to therapeutic phages. These findings indicate that bacterial genome assemblies may harbor lytic phages of potential therapeutic value [19]. A well-known example concerns phage therapy for *Mycobacterium abscessus* infection: among more than 10,000 phages tested, only one was found to be strictly lytic. Two temperate phages were genetically modified to inactivate their lysogeny genes and were successfully administered to a patient, representing a significant advance in the management of this drug-resistant infection [20]. Nevertheless, the long-term safety and regulatory acceptability of such genetically modified temperate phages remain debated, particularly regarding their stability, potential for horizontal gene transfer, and acceptability across different jurisdictions. This debate reflects a broader uncertainty about how to balance therapeutic innovation with biosafety concerns.

Another key criterion is to ensure that phage genomes are free of virulence and antibiotic resistance genes. This requires complete genome sequencing of each candidate, followed by detailed bioinformatic annotation. However, the prediction of virulence factors remains limited, as it largely depends on sequence homology with characterized elements from databases like the Virulence Factors Database [21]. As of today, despite significant progress in annotation tools, an estimated 65% of phage-encoded proteins still lack reliable functional annotation [22]. In contrast, the screening of antibiotic resistance genes is more robust, thanks to well-established databases such as ResFinder and the Resistance Gene Identifier [23,24]. In practice, the prevalence of resistance genes in phage genomes appears to be extremely low, suggesting that the risk of direct dissemination through phages remains minimal, although continued vigilance is warranted.

### 1.3. Key In Vitro Functional Parameters Relevant for In Situ Efficacy

Phage selection also relies on the in vitro assessment of functional parameters that are commonly used as initial screening criteria, as they provide insight into phage-host interactions under controlled conditions. Among these, replication kinetics and host range are essential to ensure rapid and targeted antibacterial activity.

In vitro assessment of a phage’s propagation capacity is necessary to confirm that it possesses a sufficiently high burst size (the average number of virions released per infected cell) and a relatively short latent period (the time between phage adsorption and the onset of host cell lysis) [25]. These properties support rapid amplification of the phage population under controlled conditions, thereby increasing the likelihood of reaching initially untargeted bacterial cells. Such parameters are typically determined using the one-step growth curve assay, a classical method developed in the 1930s by Ellis and Delbrück [26]. It should be noted that, although widely adopted in vitro, these parameters have limited predictive value when applied to in vivo or in situ contexts. This limitation highlights the need to complement in vitro screening with more integrative approaches capable of capturing the complexity of host-associated environments and microbial ecosystems.

Phage efficacy also depends on its ability to infect a sufficient number of strains within the target bacterial species. A host range that is too narrow may restrict its applicability to infections caused by highly clonal or genetically homogeneous pathogens. This limitation can be addressed by formulating cocktails of multiple phages selected for their complementary host ranges, thereby enabling broader coverage of the target species. Moreover, such phage combinations contribute to reducing the risk of resistance development.

## 2. Overview of Current Phage Applications

**Figure 1:**
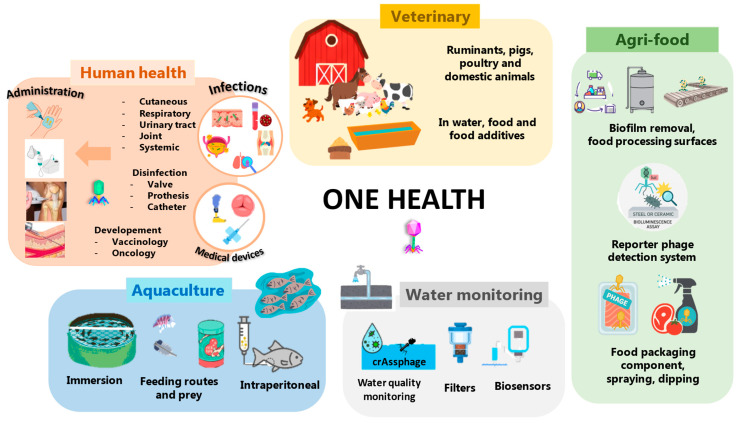
Major application domains of bacteriophages within the one health framework, including human, veterinary and aquaculture health, environmental applications and the agri-food sector.

The use of bacteriophages is increasingly embedded in a One Health framework (Figure 1), which recognizes the interdependence of human, animal, and environmental health, and promotes coordinated, interdisciplinary actions to achieve optimal health outcomes for all [27]. A non-exhaustive overview of current phage applications across these sectors is presented below to illustrate the diversity and relevance of their use in this integrative context.

### 2.1. Human Phage Therapy and Clinical Innovations

In response to the global public health crisis posed by antibiotic resistance, bacteriophages are regaining attention as either an alternative or a complement to antibiotic therapies. Their use in human medicine currently falls within two main frameworks: compassionate use, for patients with no other therapeutic options, and controlled clinical trials aimed at assessing their efficacy according to rigorous medical standards [28].

One of the most extensively studied applications is the treatment of infections caused by multidrug-resistant bacteria such as *Staphylococcus aureus*, *Pseudomonas aeruginosa*, and *Escherichia coli*, which are implicated in respiratory, urinary, cutaneous, articular, or systemic infections. For instance, in a murine model of pulmonary infection with multidrug-resistant *P*. *aeruginosa*, inhaled administration of a dry powder phage preparation led to a 5.3-log reduction in pulmonary bacterial load [29]. Moreover, combining phages with antibiotics has shown improved therapeutic efficacy in complex or persistent infections. A recent clinical study demonstrated that bacterial eradication was 70% less frequent in the absence of concomitant antibiotic administration. This synergistic effect between phages and antibiotics was observed in 90% of patients evaluated [30]. Similarly, complete eradication of a chronic *P*. *aeruginosa* prosthetic joint infection was achieved by combining phage Pa53 with the antibiotic meropenem [31]. This synergy is largely attributed to the ability of phages to penetrate and disrupt biofilms, structured bacterial communities highly resistant to antibiotics, which provides a key advantage in treating device-associated infections (e.g., catheters, joint prostheses, cardiac valves). For example, a two-phage lytic cocktail targeting *Proteus mirabilis* reduced biofilm mass by 65% in an artificial bladder model [32]. In this context, phage-derived enzymes such as endolysins are also being explored as complementary antimicrobial tools. Several recombinant endolysins have shown strong activity against *Staphylococcus aureus* biofilms, either alone or in combination with antibiotics, highlighting their potential to enhance the treatment of biofilm-associated infections [33]. In parallel, phages are increasingly investigated as precision tools for microbiome modulation. A study demonstrated that phages specifically targeting cytolysin-producing *Enterococcus faecalis* significantly reduced hepatocyte death and ethanol-induced liver damage in a humanized mouse model colonized with fecal microbiota from patients with alcoholic hepatitis. Importantly, these phages selectively eliminated pathogenic strains without disrupting the broader gut microbiota, offering a promising strategy for targeted microbiota editing in microbiome-associated diseases [34].

Phages have proven effective through multiple ways of administration, including intra-articular injection [31], intravenous infusion [20], inhalation using nebulization [35], and topical application in hydrogels [36]. Interestingly, certain phages have also demonstrated the ability to enter human cells such as macrophages and epithelial cells, and to infect intracellular pathogens like *Mycobacterium abscessus* [37], which are typically inaccessible to conventional antibiotics.

Finally, phages are being explored in innovative fields such as vaccinology and oncology. As vectors, they can be genetically engineered to display viral or tumor-associated antigenic epitopes, thereby inducing targeted immune responses. This approach is being investigated for the development of both prophylactic and therapeutic vaccines. Additionally, phages are studied for their ability to deliver therapeutic genes, cytotoxic agents, or immunomodulators specifically to tumor cells, thus contributing to novel strategies in gene therapy and cancer immunotherapy [38].

### 2.2. Animal Health and Veterinary Applications

In the veterinary field, the potential of bacteriophages as sustainable alternatives to antibiotics has also been explored in poultry, swine, cattle, and sheep [39]. These animals, often reservoirs of zoonotic pathogens, represent strategic targets for reducing the risk of transmission to humans. Used as feed additives, phages can not only control bacterial infections (e.g., *Salmonella*, *Staphylococcus aureus*, *E*. *coli*, *Campylobacter*, *Clostridium perfringens*), but also improve gut microbiota composition by promoting the growth of beneficial bacteria [40]. Several veterinary phage-based products are already available, such as BioTector^®^ S1 in poultry farming, targeting *Salmonella*. Other formulations, such as INT-401™ and PLSV-1™, are under development to combat *Clostridium perfringens* and *Salmonella* spp., respectively [41].

In poultry, studies have shown that phages administered through feed or drinking water effectively reduce intestinal pathogen loads. For instance, the SalmoFREE^®^ phage cocktail, tested on thousands of broiler chickens, significantly reduced *Salmonella* incidence without affecting growth performance or mortality [42]. Additionally, encapsulation techniques enhance phage resistance to gastric conditions and allow targeted release in the intestine [43,44]. A significant regulatory milestone was reached in July 2025, when the European Commission granted the first EU-wide authorization for a phage-based feed additive targeting *Salmonella enterica* in poultry. Developed by Proteon Pharmaceuticals SA and intended for use in drinking water, this product’s approval marks a key step toward the integration of phage applications into routine livestock practices in Europe [45].

In swine, phage cocktails targeting *E*. *coli*, *Salmonella*, and *Staphylococcus aureus* have been shown to (i) improve piglet growth, (ii) reduce diarrhea, and (iii) strengthen intestinal health by decreasing inflammation and preserving epithelial junction integrity [46,47]. Moreover, a phage hydrogel led to a 90% reduction of *Acinetobacter baumannii* in a porcine ex vivo skin wound model after just 4 h of treatment [48].

In ruminants, a combination of lytic phages and probiotics (*Lactobacillus plantarum*) demonstrated in vitro synergistic antimicrobial activity against *Staphylococcus aureus* isolated from bovine mastitis cases [49]. Phage treatments have also significantly reduced *E*. *coli* populations in calf feces and helped prevent neonatal diarrhea [50]. Other studies reported a reduction in *Salmonella* in cattle feedlots following the application of phages to pen surfaces, thereby reducing the risk of carcass contamination at slaughter [51].

Finally, in companion animals, a clinical study using a phage-based treatment for chronic otitis externa caused by *Pseudomonas aeruginosa* in dogs showed a 30% reduction in clinical symptom scores within 48 h. At 18 months post-treatment, six of the seven monitored dogs were considered cured [52].

### 2.3. Food Safety in the Agri-Food Industry

The agri-food industry faces persistent microbial risks, particularly from foodborne pathogens such as *Listeria* spp., *Salmonella* spp., *Campylobacter* spp., *Shigella* spp., and *Escherichia coli*, which can lead to severe foodborne illnesses. Bacteriophages have demonstrated efficacy across the entire food production chain, from pathogen detection to food preservation [53]. Among the most innovative applications is the use of reporter phages for rapid pathogen detection by bioluminescence, as exemplified by the PhageDx™ Listeria Assay [54]. This test relies on the specific infection of *Listeria* spp. by genetically modified phages expressing a luciferase gene, enabling highly sensitive detection in less than 25 h on industrial surfaces.

Beyond detection, phages are also employed to reduce pathogen persistence and biofilm formation on food processing surfaces. For instance, the commercial phage Listex P100 has shown significant efficacy in eliminating *Listeria monocytogenes* biofilms, with reductions ranging from 3.5 to 5.4 log CFU/cm^2^ depending on biofilm maturity [55]. Similar effects were observed on *E*. *coli* biofilms formed on stainless steel and plastic surfaces when treated with the phage phT4A [56].

Phages are also being explored as active agents in antimicrobial food packaging, by immobilizing them on the surface of polymer films. For example, a film incorporating phage T4 achieved more than a 2-log reduction in *E*. *coli* compared to untreated controls [57]. More advanced approaches involve the self-assembly of lytic phages into microgels, which can be applied as antimicrobial sprays. This strategy has shown strong efficacy against multidrug-resistant *E*. *coli* O157:H7 on red meat and other fresh products [58]. More conventional applications include phage-based dipping or spraying solutions to eliminate common foodborne pathogens from a variety of food products, as reported by Hyla et al. [59]. Several commercial products have been developed targeting *Salmonella* spp., *E*. *coli* O157:H7 and other STECs, *L*. *monocytogenes*, and *Shigella* spp. [60].

In parallel, phage-derived enzymes are also being explored for surface decontamination in food processing environments. For example, the recombinant lysin LysCSA13 achieved up to 90% reduction in *Staphylococcus aureus* biofilms on stainless steel, glass, and polystyrene surfaces [61,62].

### 2.4. Control of Infections in Aquaculture

The effectiveness of bacteriophages as biocontrol agents has been demonstrated against numerous pathogens, including *Vibrio*, *Pseudomonas*, *Aeromonas*, and *Flavobacterium*, using different administration methods such as immersion, oral delivery through feed, and intraperitoneal injection. In rainbow trout fry infected with *Flavobacterium psychrophilum*, a cocktail of two phages (FpV4 and FPSV-D22) was tested and only intraperitoneal injection significantly increased survival rates [63]. Conversely, in another study, the phage AhFM11 (Straboviridae), tested against *Aeromonas hydrophila* in *Labeo rohita*, was highly effective by all three administration methods: immersion, oral, and injection, resulting in survival rates of 95%, 93%, and 100%, respectively [64]. Thus, the efficacy of each administration method may vary depending on the pathogen targeted, the host species, and experimental conditions [65]. A complementary approach involves enriching live feed organisms, such as *Artemia salina*, with phages prior to their distribution to fish larvae or juveniles. In this case, *Artemia* enriched with phage ETP-1, targeting *Edwardsiella tarda*, enabled rapid dissemination of the phage throughout the zebrafish organism. Phage persistence was confirmed in the spleen, kidney, liver, and intestine, with no detectable immunological or histological adverse effects [66].

### 2.5. Wastewater Monitoring and Treatment

In the context of increasing water scarcity and the growing threat of antimicrobial resistance, wastewater treatment plants have emerged as strategic sites for both surveillance and intervention [67]. Within this framework, bacteriophages are garnering attention as biocontrol agents targeting multidrug-resistant bacterial pathogens. For example, in an experimental biofiltration system fed with synthetic stormwater effluent, the introduction of specific phages into granular activated carbon filters resulted in a reduction of more than to 99.9% in *Pseudomonas aeruginosa* concentrations in the filter effluent without impacting beneficial nitrifying bacteria involved in ammonium oxidation [68]. Phages are also being explored as a solution to prevent membrane biofouling, a common issue in water treatment caused by biofilm formation or the proliferation of filamentous microorganisms. Several studies have demonstrated that phages can effectively target such bacteria, including *Delftia tsuruhatensis*, *Gordonia*, and *Sphaerotilus natans*, leading to biofilm degradation and restoration of system performance, without disrupting beneficial microbial communities [69]. In parallel, phages are increasingly used for water quality monitoring, serving as indicators of human fecal contamination or as tracers for treatment efficacy. One of the most promising is the crAssphage (cross-assembly phage), which can be detected and quantified with qPCR [70,71]. Due to its high specificity for human fecal microbiota, consistent abundance, and strong stability against physical and chemical treatment processes [72], crAssphage fulfills the Bonde criteria for a robust microbial water quality indicator [73]. Monitoring its presence could therefore help assess the effectiveness of disinfection processes and membrane filtration technologies, as a proxy for enteric virus reduction [74]. Finally, nanostructured electrochemical biosensors using immobilized phages on electrodes have shown high sensitivity and specificity for rapid pathogen detection. For instance, a phage-based electrochemical sensor successfully identified *Staphylococcus arlettae* in river water in under 2 min, with a detection limit as low as 2 CFU·mL^−1^ [75].

## 3. Phage-Based Biocontrol of Phytopathogenic Bacteria: A Promising Potential for Agriculture

Alongside their expanding role in other One Health sectors, bacteriophages are receiving growing attention in agriculture for the biocontrol of phytopathogenic bacteria. Nevertheless, this field presents specific challenges related to environmental variability and the complexity of plant pathosystems.

### 3.1. Evidence of Efficacy and Key Success Factors for Phage Biocontrol

The scientific literature has seen a rapid increase in studies exploring the potential of phage-based biocontrol across a wide range of plant pathosystems. Conducted under controlled conditions or in open-field trials, these studies have helped identify several parameters that influence treatment success: phage concentration, application frequency, formulation, and the specificity of the plant compartment targeted (e.g., phyllosphere or rhizosphere). Table 1 summarizes a selection of case studies across agricultural contexts to highlight the variables contributing to successful phage-based biocontrol. Most treatments, either preventive or curative, have employed foliar spraying as the primary mode of application. While this strategy effectively targets bacteria on the leaf surface, it may be insufficient to provide durable control of pathogens residing in other ecological reservoirs such as seeds or soil.

In light of these influencing factors, recent research has explored ways to optimize delivery methods and maximize phage efficacy in complex plant pathosystems. This has led to the development of more diversified application strategies, as detailed below.

#### 3.1.1. Diversifying Application Strategies

Given the diverse reservoirs involved in the pathogen life cycles, phage-based biocontrol may benefit from an integrated approach targeting multiple stages of infection. Phage cocktails could be applied at several points along the crop production chain: seed treatment, incorporation into irrigation water, foliar spraying, and post-harvest treatment, ensuring continuous protection [81].

Seed transmission of bacterial pathogens has been well documented [103]. For example, in *Xanthomonas hortorum* pv. *vitians*, contamination may arise either from reproductive organs colonized by epiphytic bacteria or through systemic vascular infection leading to direct seed colonization [104]. In this context, phage-based seed treatments have shown that phages can remain infective around the seeds for extended periods, even post-germination. Promising results have been obtained against seed-borne pathogens such as *Acidovorax valerianellae* and *A*. *citrulli*, causal agents of black spot in lamb’s lettuce and bacterial fruit blotch in cucurbits, respectively. Seed coating with phages resulted in up to 87% reduction in bacterial populations, while simultaneously increasing germination rates from 59% to 93% [85,105].

Other environmental reservoirs such as soil, weeds, and irrigation water, can also serve as sources of bacterial inoculum. Using phages in irrigation water has shown potential, especially against soilborne pathogens like *Ralstonia solanacearum*, which infects plants via the roots. Álvarez et al. [76] demonstrated that phage irrigation treatment significantly reduced bacterial loads and disease incidence under field like conditions. Moreover, the phages remained viable in water for several months, suggesting adequate persistence for agricultural use.

In post-harvest settings, conventional decontamination practices typically involve chlorinated water to minimize bacterial adaptation during storage [106]. As a more sustainable and less harmful alternative, phage-based treatments have been explored. For instance, combining phages with gaseous ozone on spinach leaves produced a synergistic effect, reducing *E*. *coli* O157:H7 populations by up to 5.2 log CFU·g^−1^ [107]. Similarly, commercial phage products targeting *Listeria monocytogenes* were applied to iceberg lettuce leaves without compromising sensory quality (taste, texture, visual appearance), confirming compatibility with industrial standards [108].

Overall, these studies demonstrate that diversifying application strategies across different plant compartments and crop stages enhances the likelihood of successful phage-based biocontrol. Such integrative approaches are essential to overcome the limitations of single-point interventions and improve treatment robustness in real-world agricultural settings.

#### 3.1.2. Optimization of Phage Application Methods and Formulations

Despite numerous reports demonstrating the effectiveness of bacteriophages in controlling phytopathogenic bacteria, their large-scale deployment in agriculture remains limited by several constraints. The main constraint among these is the stability of phages within the various plant holobiont compartments, particularly the phyllosphere.

The phyllosphere represents a particularly hostile environment for phages, exposing them to desiccation, fluctuating climatic conditions, and especially UV radiation, which is recognized as the most detrimental factor. For example, following foliar application of phage ΦXacm 2004-16 targeting *Xanthomonas axonopodis* pv. *citrumelo*, no detectable phage particles remained after 4 to 10 h, depending on the time of day [109]. Lang et al. reported more encouraging results, detecting phages targeting *X*. *axonopodis* pv. *allii* up to 5 days in greenhouse and 4 days in open field conditions but with a rapid titer decline of two logarithmic units was observed within just 12 h [110]. To improve phage persistence in the phyllosphere, several strategies have been investigated, generally falling into three categories: biological, formulation-based, and agronomic practices.

One of the promising biological strategies involves the use of “carrier” bacteria that are both avirulent and closely related to the target pathogen, and that can stably colonize the plant surface while remaining susceptible to the phages. These strains act as auxiliary hosts, supporting phage replication directly on the plant and helping to extend their persistence. This approach was tested against *Erwinia amylovora* using the non-pathogenic epiphytic bacterium *Pantoea agglomerans*, a close relative of the pathogen. Co-application of phage ΦEa2345-6 and the carrier strain reduced floral symptom severity by 56% in potted plants [102]. Similarly, co-application of avirulent *Xanthomonas* strains and phages significantly increased phage persistence on leaves for up to 3 days in both greenhouse and field conditions. This approach also improved disease control in field trials, achieving results comparable to copper sulfate treatments [111,112].

Various additives have been tested to improve the effectiveness of phage applications on plant surfaces. Among them, certain adjuvants such as stickers, wetting agents, or UV-protectants can (i) mitigate abiotic stresses, (ii) enhance droplet adhesion by reducing surface tension, and (iii) promote more uniform distribution across the leaf surface [113]. These components are often incorporated into more complex formulations that also include stabilizers and nutrient sources. Historical examples include: (i) 0.5% pregelatinized corn flour with 0.5% sucrose; (ii) 0.75% skim milk with 0.5% sucrose; and (iii) 0.5% Casecrete NH-400 with 0.5% sucrose and 0.25% corn flour. Such mixtures have shown greater phage persistence and better disease suppression in field trials against *Xanthomonas campestris* pv. *vesicatoria*, compared to phages applied without formulation [114]. Phages formulated with Casecrete NH-400 persisted over 1000 times longer than unformulated phages 36 h post-application [114]. More recent studies on *X*. *oryzae* pv. *oryzae* confirmed the efficacy of simple additives such as skim milk and rice or corn flour, which preserved phage titers and significantly reduced disease severity under greenhouse conditions compared to unformulated phages [115].

A wide range of natural, UV-absorbing compounds that are readily available, non-toxic, and suitable for agricultural use have been tested to improve phage survival. These include food-grade substances such as carotenoids (for example, astaxanthin), vegetable juices like carrot, beetroot, and red bell pepper, aromatic amino acids such as phenylalanine, tryptophan, and tyrosine, as well as casein, peptones, and Tween 80. All improved in vitro persistence of *E*. *amylovora* phage Y2 in a dose-dependent manner, with higher juice concentrations providing better protection [116]. More recently, several individual compounds and formulations were evaluated in vivo in kiwi orchards for their protective effect on phages targeting *Pseudomonas syringae* pv. *actinidiae*. Among them, the 2% casein formulation was most effective, maintaining phage titers within ~1 log reduction over 100 h, whereas unformulated phages were undetectable after 48 h [117]. However, certain formulations may also have unintended effects. In the previous study, 0.5 M sucrose attracted insects to treated plants. Additionally, while some adjuvants like skim milk enhance phage survival, they may promote pathogen growth by supplying carbon and nitrogen, highlighting the importance of testing each formulation independently on the pathosystem [101]. Formulation efficacy is also likely phage-specific. For instance, Tween 80 was significantly more protective for *E*. *amylovora* phages than for *P*. *syringae* pv. *actinidiae*, improving survival by 78% compared to control [117,118].

Recently, other advanced formulation strategies have been explored, including encapsulation of phages in nanomaterials such as liposomes, sodium alginate, or more complex systems combining chitosan, alginate, and CaCl_2_. Lipid nanocarriers and nanofibers have also shown promise. While this technology is already proven in health and food sectors, it is now being adapted for phage biocontrol [119,120]. In a recent study, Choudhary et al. [121] formulated phage ΦXp06-02-1 (targeting *X*. *perforans*) with manganese-doped zinc sulfide nanoparticles coated in N-acetylcysteine. Post sunlight exposure, this formulation increased phage persistence in the phyllosphere 15-fold and reduced disease severity by 16.4% compared to unformulated phages.

Selecting appropriate excipients for phage formulations remains challenging. While certain compounds commonly reported in the literature, such as skim milk, sucrose, and casein, can enhance phage stability under UV exposure, others may reduce infectivity by disrupting phage adsorption to the bacterial host. This inhibition may result from non-specific interactions with bacterial receptors or from direct adsorption of phage particles onto excipient components. It is therefore essential to systematically evaluate the compatibility of each phage within a cocktail with the intended formulation. Such evaluations should include assessments of (i) phage stability after formulation, (ii) potential phytotoxicity, and (iii) actual persistence on the phyllosphere.

Finally, application method also significantly impacts phage treatment efficacy. Balogh et al. recommend evening applications to minimize UV degradation and extend phage–host interaction time [114]. As with conventional pesticides, it is estimated that less than 1% of the active substance actually reaches its biological target [122]. Major losses are due to droplet dispersion, evaporation, runoff, and rain wash-off [123,124]. Emerging technologies, such as AI-assisted “smart nozzles” guided by real-time image recognition, may enable precise targeting of vegetated areas. One example is SpotSprayING, developed by Farm-ING Smart Farm Equipment (FlexCo), which activates nozzles only upon vegetation detection. Thoughtful integration of these various strategies will be essential to optimize the effectiveness of phage-based biocontrol in agricultural contexts.

Taken together, these findings underscore the importance of optimizing both the formulation and delivery of phages to improve their persistence and activity in variable field conditions. While many adjuvants and carriers show promise, context-dependent evaluations remain essential to ensure phage viability, compatibility with target crops, and biosafety.

#### 3.1.3. Incorporating Phages into an Integrated Disease Management Approach

To enhance the effectiveness of phage-based biocontrol, an integrated pest management approach appears particularly promising. However, few studies have explored the additive or synergistic effects of combining phages with other biocontrol agents, such as PGPR (Plant Growth-Promoting Rhizobacteria), antimicrobial peptides, or plant defense elicitors. Each of this biocontrol relies on distinct mechanisms and has its own limitations, including limited environmental stability, narrow spectrum of action, or high production costs. Nonetheless, their rational combination could improve overall efficacy while minimizing the risk of resistance development.

PGPR, especially *Pseudomonas* spp., are among the most studied alternatives to chemical inputs [125]. Their biocontrol potential is based on both direct effects such as the production of antimicrobial metabolites (antibiotics, bacteriocins, siderophores) and indirect effects through the induction of systemic resistance (ISR) in plants [126]. Some studies have shown functional complementarity between phages and PGPR. For instance, a mutation in LPS that conferred resistance to phages also increased bacterial susceptibility to antibiotics produced by *Bacillus amyloliquefaciens* [127].

Antimicrobial peptides represent a second strategic approach. Peptaibols derived from Trichogin GA IV have notably demonstrated both in vitro and in planta inhibitory activity against *Xanthomonas campestris* pv. *campestris* by disrupting the bacterial membrane. However, two main limitations hinder their application in biocontrol: (i) high production costs due to complex synthesis methods, and (ii) potential phytotoxicity, with certain peptides causing significant plant mortality at concentrations of 50 µM in tobacco [128]. Combining these peptides with phages could partly overcome these constraints by lowering the required doses, thereby reducing both toxicity risks and treatment costs.

The combination of plant defense elicitors and phages has also shown efficacy in certain pathosystems. For example, acibenzolar-S-methyl applied with phages resulted in better disease protection than phages alone against *X*. *campestris* pv. *vesicatoria* and *X*. *axonopodis* pv. *allii* [110,129].

This combined strategy could also enable broader biocontrol activity, including against fungal pathogens not initially targeted. Certain bacterial phytopathogens, such as *X*. *hortorum* pv. *vitians*, may facilitate secondary infections by fungi or oomycetes [130]. Reducing bacterial populations on the phyllosphere may thus limit infection sites and pathogen entry into plant tissues. This approach could enhance the robustness and durability of biocontrol, broaden its spectrum, and mitigate the risk of resistance emergence.

Overall, integrating phages with complementary biocontrol tools could offer more robust, sustainable, and context-adapted solutions to manage bacterial plant diseases.

### 3.2. Initial Deployment and Commercialization of Phage-Based Biocontrol

Several commercial phage-based products have already been approved in specific countries, marking an important step in the transition from laboratory research to field application of these biological agents.

In the United States, the Environmental Protection Agency has approved multiple phage-based formulations as biological pesticides. The Agriphage™ line, developed by Omnilytics Inc. (USA), was among the first industrial initiatives in this domain, with initial approval granted in 2006. It now includes five products targeting key phytopathogens of major crops, such as tomatoes, citrus, stone and nut fruits, and pome fruits: spot & speck (against *Xanthomonas* spp. and *Pseudomonas syringae* pv. *tomato*), tomato canker (against *Clavibacter michiganensis* pv. *michiganensis*), fire blight (against *Erwinia amylovora*), citrus canker (against *Xanthomonas citri* pv. *citri*), nut & stone fruit, targeting *Xanthomonas arboricola* pv. *pruni*, *X*. *arboricola* pv. *juglandis*, *X*. *arboricola* pv. *corylina*, and *P*. *syringae* pv. *syringae*.

More recently, XylPhi-PD™ (developed by A&P Inphatec) was approved for use against *Xylella fastidiosa* in vineyards. This phage cocktail is applied via direct injection into the vine’s xylem.

In Europe, approvals remain limited. APS Biocontrol Ltd. (UK) markets the patented Biolyse^®^ technology, focusing on developing phage cocktails for crop protection, particularly against *Pectobacterium* spp., and post-harvest applications targeting pathogens on ready-to-eat lettuce and cultivated mushrooms. In Hungary, Enviroinvest Corp. developed Erwiphage PLUS^®^, the first phage-based biopesticide authorized in Europe. It targets *Erwinia amylovora*, the causal agent of fire blight in apple and pear trees. This product received temporary national authorization in 2012 and has been periodically renewed. The company is also working on new phage cocktails targeting *X*. *arboricola* pv. *juglandis* (walnut blight) and *X*. *oryzae* pv. *oryzae* (bacterial leaf blight of rice).

These products, which target various bacterial pathovars on vegetable, fruit, and field crops, demonstrate the practical feasibility of agricultural phage use, even within a still-fragmented regulatory framework. However, their broader deployment remains constrained by key scientific challenges, particularly the risk of bacterial resistance emerging under field conditions.

### 3.3. Emergence of in Planta Resistance and Cocktail Stability

One of the main challenges associated with the use of phages for biocontrol is the emergence of resistant bacterial mutants. The limited number of studies conducted under natural conditions using phage cocktails have shown contrasting outcomes. In *Pseudomonas syringae* pv. *tomato* and pv. *syringae*, despite extensive phyllosphere sampling, no resistance was detected neither in in planta coevolution experiments nor during orchard field trials [131,132]. In contrast, resistant mutants of *Ralstonia solanacearum* were isolated from the rhizosphere after cocktail application; however, these mutants displayed reduced growth rates and diminished competitiveness [133].

Beyond the emergence of resistance, the long-term genetic stability of phage cocktails remains a critical concern. To date, no study in the agricultural sector has investigated the genetic dynamics of phage cocktails in planta, representing a significant knowledge gap in assessing their durability under real-world conditions. In contrast, studies in the medical field have documented genetic recombination events within receptor-binding regions between distinct phage genera, such as *Litunavirus* and *Luzseptimavirus*, during phage training experiments with *Pseudomonas aeruginosa* [134]. While this genetic plasticity may facilitate phage adaptation, it also underscores the need for systematic monitoring of the genetic stability of applied phages to evaluate potential evolutionary changes and their consequences for host range and efficacy.

#### Evolutionary Trade-Offs Associated with Phage Resistance and Implications for Biocontrol

Although the emergence of phage-resistant bacterial mutants is considered a potential threat to the long-term efficacy of phage treatments, the mutations involved may paradoxically be advantageous for biocontrol. Several studies in diverse contexts have highlighted pleiotropic effects associated with resistance, often resulting in reduced virulence, competitive fitness, or environmental survival of resistant strains [135]. These evolutionary trade-offs typically arise from the loss or modification of bacterial surface structures that serve both as phage receptors and virulence factors such as flagella or lipopolysaccharides (LPS) [136].

For instance, in *Xanthomonas oryzae* pv. *oryzae*, out of 19 mutants resistant to phage X2, three exhibited a significant reduction in virulence, with an average 84% decrease in lesion length, 48% reduction in swimming motility, and 42% decrease in biofilm formation. Some of these mutations were located in genes involved in LPS biosynthesis (e.g., glycosyltransferases, core oligosaccharide synthesis) [137]. Similarly, among 36 genes identified as essential for infection by phage Φ*Xhv*-1 in *Xanthomonas hortorum* pv. *vitians* [138], 23 overlapped with genes previously found to contribute to in planta bacterial fitness [139], including loci within *cps*-*rml* and LPS regions 1 and 2. Mutants in these regions all exhibited ~50% symptom reduction compared to the wild-type strain [138]. In *Burkholderia glumae*, phage S13-resistant mutants displayed mutations in genes encoding structural components of the flagellum (e.g., *flgA*, *flhC*, *flgC*, *flgG*, *flgK*). These mutants completely lost swimming motility in vitro, and deletion mutants (Δ*flgC* and Δ*flgK*) no longer caused symptoms in rice seedlings. However, some point mutants in *flgC* and *flgF* retained virulence comparable to the wild-type. The authors suggest possible genetic reversion during rice germination or growth, with in planta selective pressures potentially promoting partial recovery of motility and virulence, complicating the dynamics of the resistance–virulence trade-off [140].

In the context of disinfecting nutrient solutions in hydroponic greenhouses using phages targeting *Agrobacterium* biovar 1, Fortuna et al. [141] isolated resistant *Agrobacterium* mutants following treatment with phage OLIVR4 or OLIVR5. Mutations associated with OLIVR4 were located in genes involved in LPS biosynthesis, whereas those linked to OLIVR5 affected genes related to the regulation of surface factor expression (*rcsC*), membrane efflux of toxic compounds (*acrB*), and a gene encoding ferredoxin NADP^+^ reductase. These alterations suggest potential indirect effects on the expression or accessibility of the flagellar receptor. Phenotypically, only OLIVR4 mutants showed strong reductions in swimming motility. However, all mutants retained full virulence in a hairy root disease model in bean, indicating no trade-off. In *Pectobacterium carotovorum* subsp. *carotovorum*, Kim et al. [142] showed that clones resistant to a three-phage cocktail displayed more pronounced phenotypic alterations compared to those exposed to a single phage, suggesting cumulative deleterious effects resulting from mutations selected under multi-phage pressure. Therefore, phage cocktails, by applying multi-target selective pressure, may not only reduce the risk of cross-resistance but also amplify the adaptive costs of resistance, steering bacterial evolution toward less competitive and less virulent phenotypes.

Altogether, these results challenge the assumption that phage resistance in planta necessarily undermines biocontrol performance. On the contrary, resistance mutations often come with biological trade-offs that reduce pathogen virulence or competitiveness effects that may indirectly support disease management over time. Moreover, phage cocktails applying diverse receptor pressures can increase the evolutionary cost of resistance, potentially limiting its emergence and persistence. However, the absence of systematic in planta monitoring of phage evolution and cocktail dynamics under field conditions remains a significant gap. Moving forward, a more integrated ecological perspective is needed to assess how phages and their lytic activity may impact not just pathogens, but also the broader plant-associated microbiome and soil ecosystem.

### 3.4. Expanding the Evaluation of Phage Biocontrol to the Plant–Soil Ecosystem and Biogeochemical Cycles

To assess the effectiveness of phage applications in biocontrol, most published studies to date have relied on three main criteria: (i) the reduction in disease symptoms, (ii) the decrease in the population of the targeted pathogen, and (iii) more rarely, the durability of the treatment, estimated by the absence of spontaneous emergence of phage-resistant bacterial mutants. While these indicators are essential for evaluating the success of a phage-based biocontrol approach, they only reflect a limited part of the potential impacts that phages may have within the broader plant–soil ecosystem.

#### 3.4.1. Modulation of Plant Metabolism and Defenses by Phages and Virocell Cecromass

During interactions with phytopathogenic bacteria, plants undergo profound metabolic reprogramming, characterized by the accumulation of sugars and amino acids required for maintaining primary metabolism and for synthesizing secondary defense compounds [143]. Although phages are primarily studied for their antibacterial effects, several recent studies suggest that they may also influence plant metabolism, either directly or indirectly. In particular, the biological material released during bacterial lysis, known as virocell necromass, is far from inert. It may alter the plant’s metabolome and thereby impact plant health. In the rhizosphere, Novak et al. [144] demonstrated that the introduction of virocell necromass stimulated the production of root-derived metabolites. On the phyllosphere, Papaianni et al. [145] observed that phage treatment led to a general decrease in the accumulation of amino acids and nitrogen-containing compounds, while citrate strongly accumulated, suggesting a metabolic shift toward the Krebs cycle and ATP production. In the same study, phage application did not result in overexpression of genes associated with systemic acquired resistance. However, Skliros et al. [79] reported contrasting findings, showing increased expression of plant defense-related genes (e.g., *PR1b*, *Pin2*) following phage treatment.

Additionally, certain structural proteins of phages could potentially be recognized by plants as pathogen-associated molecular patterns, triggering immune responses. Beyond the phage particles themselves, the lysis of bacterial cells results in the release of bacterial components, such as LPS, which are well-known elicitors of plant innate immunity through Pattern Recognition Receptors. This recognition can lead to: the production of reactive oxygen species (ROS), the expression of defense genes, and the reinforcement of the plant cell wall [146,147].

#### 3.4.2. Effects of Phages and Bacterial Lysis on Microbial Communities

Phage biocontrol through irrigation or foliar spraying inevitably results in a substantial proportion of the applied phages being released into the soil. Consequently, it is also relevant to investigate the microbial response of this compartment. One of the main advantages of phages in biocontrol lies in their generally narrow host range, which enables specific targeting of bacterial pathogens and limits direct effects on non-target bacteria. However, the elimination of a microbial species that plays a central ecological role (e.g., keystone species, ecosystem engineer, umbrella species) may still have indirect consequences on the structure, composition, and dynamics of microbial communities. These effects can occur through mechanisms such as competitive interactions, niche release, or trophic cascades. In addition, the release of nutrients from lysed bacteria can influence the abundance and composition of surviving populations. It is important to note that naturally occurring phages already play a key role in shaping microbial communities [148]. In the rhizosphere, Wang et al. [149] demonstrated that repeated applications of a phage cocktail significantly modified microbial diversity and community structure, notably leading to enrichment in *Actinobacteria* and *Chloroflexi*. *Actinobacteria*, in particular, are recognized for their beneficial roles in plant health and soil suppressiveness, suggesting that phage activity could indirectly support protective bacterial communities. Such microbial shifts could therefore contribute positively to integrated pest management strategies. In the phyllosphere, Papp-Rupar et al. [150] also observed that phage treatment increased alpha diversity of the bacterial microbiome, likely due to the reduction in the dominant pathogen. Similarly, Jiang et al. [82] found that phage application against *Xanthomonas oryzae* pv. *oryzae* not only reduced the pathogen population, but also significantly altered the epiphytic and endophytic community composition, notably increasing the relative abundance of *Sphingomonas* and *Stenotrophomonas*, two genera often associated with beneficial functions in plants.

In addition to these biological effects, a recent study revealed that a substantial proportion of phages may be associated with multiple bacterial species across diverse ecosystems [6]. These findings challenge the classical view of strict host specificity and suggest that some phages may have broader interaction potential than previously assumed. However, as acknowledged by the authors, the analysis does not currently allow us to conclude that the detected associations correspond to productive infection cycles or viral particle production.

Together, these findings highlight that phage applications may influence microbial communities both directly and indirectly, through mechanisms that are not yet fully understood. This reinforces the importance of monitoring microbial population dynamics, particularly through 16S gene profiling, following phage application, to evaluate both safety and ecological impact.

#### 3.4.3. Consequences of Phage Activity on Soil Biogeochemical Cycles

Beyond their effects on microbial community composition, phages are also known to influence key functions in biogeochemical cycling, especially in marine ecosystems. Two major processes have been described in this context: the viral shunt, which enhances the rapid recycling of organic carbon and nitrogen through bacterial lysis, and the viral shuttle, which promotes the sequestration of more recalcitrant organic compounds [151]. Emerging research suggests that similar dynamics may also occur in terrestrial soils. For instance, the addition of phages in soil microcosms has been shown to increase ammonium (NH_4_^+^) concentrations, attributed to the mineralization of organic nitrogen released during bacterial lysis [152]. In another study, phage application stimulated microbial respiration, fueled by labile organic compounds released through the viral shunt, while simultaneously promoting greater carbon sequestration through the viral shuttle pathway, resulting in the formation of more recalcitrant soil organic matter [153]. Furthermore, these processes may help alleviate nutrient limitations for soil microorganisms, particularly phosphorus limitation, by increasing the pool of dissolved organic matter, thus supporting microbial activity and ecosystem functioning.

Altogether, these results highlight that phage applications can influence soil functioning and nutrient cycling, placing them at the interface between crop protection and agroecosystem management. This dual role raises important questions regarding how phage-based products should be evaluated, regulated, and integrated into agroecological transition strategies.

## 4. Phages and the Agroecological Transition: A Pesticide Alternative in the Face of Regulatory Barriers

Globally, the agricultural biocontrol market is booming, with an estimated potential value of €50 billion. Tree crops, vineyards, and vegetable farming are among the largest users, reflecting the growing integration of these solutions into agricultural practices. In Europe, this economic momentum is supported by proactive public policy, aligning with key objectives of the European Green Deal and the Farm to Fork strategy, which aim to reduce the use of plant protection products by 50% by 2025. In this context, interest in phage-based products within the agricultural sector is rising, though currently limited to a few national frameworks. To date, no phage-based products have been approved by the European Food Safety Authority as plant protection products or biopesticides, although four dossiers are currently under evaluation (https://ec.europa.eu/food/plant/pesticides/eu-pesticides-database/start/screen/active-substances, accessed on 29 December 2025). By contrast, several phage-based products have been commercially available in the United States for years, illustrating their feasibility for large-scale agricultural deployment.

### 4.1. Regulatory Barriers in Europe: A Major Obstacle to Innovation

The current European regulatory framework for phage-based biocontrol remains largely modeled on that of conventional chemical pesticides. In the absence of a harmonized legal definition of biocontrol across Member States, phages are assessed under Regulation (EC) No 1107/2009, a procedure initially designed for synthetic agrochemicals. This framework is lengthy, complex, and costly, and is often cited as a major bottleneck for innovation in microbial biocontrol. According to professional organizations, obtaining market authorization in the EU takes 10 to 12 years on average, compared to less than 3 years in the US. In contrast, countries such as Brazil have implemented fast-track processes for low-toxicity products, organic farming application (6 months to 1 year) and emergency phytosanitary uses for minor or priority crops with limited pesticide options [154].

In this context, various institutions have acknowledged the need for reform. A 2024 report by the Joint Research Centre explicitly highlighted the limitations of the current framework and called for greater flexibility, particularly to allow the routine adaptation of phage cocktails, which is essential given the evolution of bacterial pathogen populations [155]. The OECD has also issued a dedicated guidance document on bacteriophages, offering practical recommendations for their evaluation as plant protection products [156]. In parallel, some EU Member States have resorted to temporary national authorizations, most likely under Article 53 of Regulation (EC) No 1107/2009, which permits emergency approval for up to 120 days, under strict conditions. These authorizations may be granted in cases of danger that cannot be contained by any other reasonable means. For instance, Erwiphage PLUS^®^, developed by Enviroinvest Corp. to target *Erwinia amylovora*, received such an authorization in Hungary in 2012 and has been periodically renewed since. The Scottish company APS Biocontrol markets Biolyse^®^, a postharvest aid to prevent *Pectobacterium* infection in potato tubers. While these cases illustrate the regulatory flexibility available at the national level, they also underscore the fragmented and inconsistent implementation of EU legislation, still a major barrier to broader adoption and industrial investment.

In December 2025, the European Commission proposed a legislative simplification package to revise Regulation No 1107/2009 and 369/2005. This proposal aims to clarify the definition of biopesticides, enable provisional authorizations based on draft assessments, and prioritize the evaluation of biocontrol agents. Although still under discussion, this initial represents a potential shift toward more supportive regulatory pathways for biological solutions, including phages. The International Biocontrol Manufacturers Association (IBMA) welcomed the proposal, while calling for clear timelines and harmonization across Member States.

Finally, intellectual property issues remain an additional barrier. Natural phages cannot be patented, which limits private investment. While engineered phages may qualify for patent protection, they raise regulatory uncertainties, particularly concerning their potential classification as generally modified organisms, further complicating their approval and public acceptance.

### 4.2. Phage-Based Biocontrol: A Societal Innovation in Agriculture

Beyond regulatory challenges, the economic and social acceptance of phage-based biocontrol remains a key issue. Often perceived as less effective and more expensive than conventional chemicals, biocontrol products raise concerns about their actual adoption by farmers. However, some commercial success stories show promising signs: for instance, 40% of potatoes sold in UK supermarkets are currently treated with APS Biocontrol’s phage formulations [157].

The diffusion of such innovations will also depend on farm structure. In monoculture systems, where a single crop accounts for the majority of farm income, investment in a targeted phage treatment may be more easily justified. Conversely, in polyculture systems, the cost-effectiveness of a phage-based suspension targeting a specific pathosystem will likely depend on the acreage and revenue of the affected crop. More broadly, the role of cooperatives, advisory organizations, and public incentive policies will be essential in supporting producers, offsetting initial costs, and promoting the environmental benefits of phage biocontrol.

**Figure 2:**
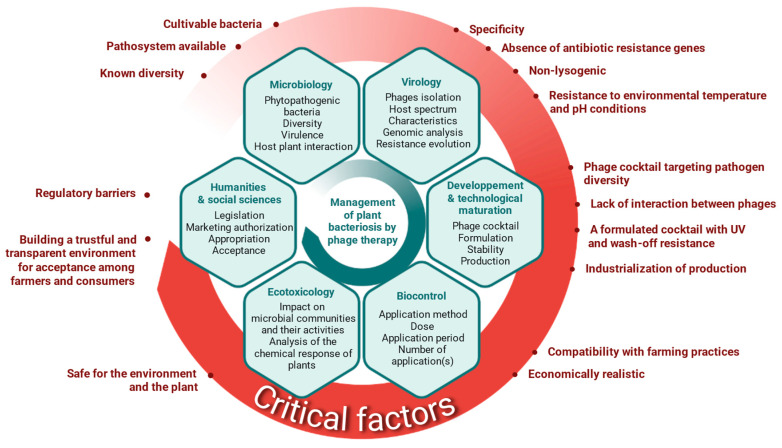
Identification of expertise and critical factors for the deployment of phage biocontrol against plant bacterial diseases.

However, social acceptability does not lie solely with producers. Consumers play a decisive role. There is strong global demand for alternatives to synthetic pesticides, especially those aligned with agroecological practices. In this context, phages may benefit from a favorable perception, as natural and environmentally friendly solutions. Still, skepticism may persist, as the term “virus”, rightly associated with phages, could negatively influence public perception in agricultural contexts. To overcome these concerns, public awareness and education efforts are crucial, grounded in scientific evidence of safety and environmental benefits. This is essential to foster their wider and long-term acceptance. Ultimately, the effective deployment of phage-based biocontrol requires an integrated approach, combining scientific, technological, regulatory, and societal expertise (Figure 2).

## 5. Perspectives

The increasing prevalence of multidrug-resistant bacterial pathogens continues to drive interest in bacteriophages as alternative or complementary antimicrobial agents across One Health domains. However, this renewed focus also brings forth new challenges, particularly concerning the evolving traits of both bacteria and their phages such as shifts in virulence, host range, and environmental persistence. Future research should aim to evaluate the capacity of phages and their bacterial hosts to persist, adapt, and exchange resistance mechanisms across interconnected ecosystems.

To meet these challenges, a deeper understanding of phage–host interactions is essential, particularly to identify bacterial surface receptors involved in both phage adsorption and virulence. Targeting such dual-function receptors could enhance therapeutic efficacy while limiting resistance emergence. In this context, the integration of artificial intelligence approaches, leveraging genomic and phenotypic datasets, holds strong potential for predicting optimal phage candidates, designing synergistic cocktails, and improving the robustness and long-term success of phage-based strategies.

At the same time, phage resistance is often mediated by complex bacterial defense systems (e.g., CRISPR-Cas, restriction-modification, abortive infection), whose distribution, diversity, and mobility remain poorly characterized in natural, clinical, and agricultural environments. Mapping and understanding these systems will be key to anticipating resistance trajectories and developing phages capable of overcoming them.

Finally, long-term in vivo monitoring of bacterial populations following phage application is crucial to track resistance dynamics and assess the ecological and evolutionary impacts of phage interventions. This includes evaluating the potential for resistant strains to disseminate across human, animal, plant, and environmental compartments which is an essential consideration for ensuring the safe, effective, and sustainable use of phages in One Health contexts.

## Figures and Tables

**Table 1:** State of the art on biocontrol by bacteriophages in plant production. For each publication that evaluated the effectiveness of phage-based biocontrol under controlled conditions (climatic chamber or greenhouse) or in the field, the following elements are summarized: the crop of interest and the associated phytopathogenic bacterium, the bacterial disease, the bacteriophages used, the application strategy (method, formulation, MOI or concentration, number of applications), as well as the main results obtained regarding disease control.

Plant	Phytopathogenic Bacterium	Bacteriosis	Phage/Cocktail	Application					Results	Reference
				Mode	Formulation	MOI ^(1)^	Number	Condition ^(2)^		
Tomato	*Ralstonia solanacearum*	Bacterial wilt	vRsoP-WF2	Watering	Water	10 to 10^4^	1	C	Decrease in wilted plants from 25% to 5% at an MOI of 10^3^.	[76]
			vRsoP-WF2 and vRsoP-WM2, and vRsoP-WR2	Watering	Water	10 to 100	1	C	Decrease in wilted plants from 50% to 0% at an MOI of 100.	
	*Ralstonia peudosolanacearum*	Bacterial wilt	RpT1, and RpY2	Watering	SM Buffer	0.1 to 10	1	C	Symptom severity was reduced by 20% and 50% using a single phage and a phage cocktail at MOIs of 1 and 10, respectively.	[77]
					4% adjuvants	10	1	C	Addition of adjuvants appears to increase treatment efficiency.	
	*Xanthomonas euvesicatoria* pv. *perforans*	Bacterial stain	PL4, S4, GF2	Foliar spraying	Water or copper hydroxide	100	1	G	Reduction in disease severity by a factor of 2 to 4.	[78]
	*Pseudomonas syringae* pv. *tomato*	Bacterial speck of tomato	Medea1	Watering the roots or foliar spraying	CaCl_2_ 1 M, MgSO_4_ 1 M	1.25 × 10^5^ PFU/mL	1 or 2	G	Disease reduction by a factor of 3 for both application modes.	[79]
Banana tree	*Ralstonia solanacearum*	Bacterial wilt	M5 and M8	Watering before plantation	TSB culture medium	1	1	G	Phages and bacteria were inoculated into the soil where healthy banana plants were then planted. Only the phage cocktail provided complete protection. Soil treatment immediately prior to planting can serve as an effective protective strategy.	[80]
Cabbage	*Xanthomonas campestris* pv. *campestris*	Black rot	FoX2 and FoX6	Watering	Water	ND–high	6	G	Only treatment with 10^9^ PFU/mL nearly eliminated the symptoms.	[81]
				Foliar spraying	0.025% Silwet Gold	0.1 to 10	1	G	Reduction in infected leaves by a factor of 4 only at a MOI of 10.	
				Foliar spraying	Water or CaCl_2_ (2 mM)	200	3 or 4	F	Decrease from 38% to 21% with the cocktail formulated in CaCl_2_.	
Rice	*Xanthomonas oryzae* pv. *oryzae*	Vascular bacteriosis	J2, J3, E	Foliar spraying	ND	10^6^ PFU/mL	1	G	Reduction in the disease index from 64.3% to 44%.	[82]
			φXOF4	Seed immersion	SM Buffer	0.001 to 10	1	G	Proportional decrease in disease severity with 10^5^ to 10^7^ PFU/mL and complete with 10^8^ PFU/mL.	[83]
Walnut tree	*Xanthomonas arboricola* pv. *juglandis*	Walnut blight	f 20-Xaj, f 29-Xaj, f 30-Xaj	Spray application	ND	ND	6	F	Disease reduction by a factor of 2 with the highest phage dose (4 cc/L) regardless of the trial season (comparable to copper treatment).	[84]
Melon	*Acidovorax citrulli*	Bacterial stain on fruit	ACPWH	Watering	ND	10^8^ PFU/g soil	1	I	Reduction in disease severity from 80% (untreated) to 27% (treated).	[85]
Potato	*Dickeya solani*	Soft rot	LIMEstone1 and LIMEstone2	Tuber immersion	Phage buffer (10 mM Tris-HCl pH 7.5; 10 mM MgSO_4_ 150 mM NaCl)	100	1	F	Reduction in disease incidence by 5% and increase in yields by 13%.	[86]
			Dagda, Mysterion, Luksen, Coodle, Kamild, Ninurta	Immersion	SM Buffer	About 100	1	I	Reduction in infected tubers from 93% to 48% with the 6 phages cocktail.	[87]
		Potato blackleg	φDs3CZ and φDs20CZ	Solution deposit	ND	10 to 100	1	G	Reduction in plant infection intensity proportionally to phage concentration. (10^8^ PFU/mL reduced by 79% to 95%, and 10^6^ PFU/mL by 46% to 51%).	[88]
				Immersion	ND	20	1	F	Reduction in infection by 87% with preventive treatment, and by 36% with curative application.	
	* Pectobacterium atrosepticum *	Soft rot	Nepra, Lelidair, Nobby, Slant, Gaspode et Momine	Tuber washing	Water	At least 100	1	I	Reduction in disease severity by 61% with the phage cocktail.	[89]
	*Dickeya* spp.	Soft rot	ΦPD10.3 and ΦPD23.1	Deposit on a slice of potato	Water	0.01	1	I	Reduction or prevention of maceration of tuberous tissues by co-inoculation of single phages or cocktail with the pathogen.	[90]
				Deposit on tuber	Water	0.01	1	I	Reduction in maceration of tuberous tissues by at least 80% same inoculations as above.	
	*Streptomyces scabies*	Common scab	ØAS1	Immersion	PYCa medium	1 × 10^9^ PFU/mL	1	G	Disease reduction on second-generation tubers from 23% to 1.2%. Demonstration of the effectiveness of phage therapy against a spore-forming bacterium.	[91]
Onion	*Pectobacterium carotovorum* subsp. *carotovorum*	Soft rot	phiMA11, phiMA12, phiMA13 and phiMA14	Immersion or spraying	Nutrient broth	0.01 to 1	1	F	Mixed results in terms of protection of bulbs planted in contaminated plots over the three years of trials with the two application modes.	[92]
Green onion	*Xanthomonas axonopodis* pv. *allii*	Bacterial leaf blight	Phi16, phi17, phi31	Foliar spraying	ND	10^−3^ to 1	1	G	Slowing of disease progression with the application of single phages or in a cocktail. Best protection with the highest concentration.	[93]
						1	4	F	Reduction in the disease index by 30% to 40% with single phages or in a cocktail.	
Vine	* Xylella fastidiosa *	Pierce’s disease	Sano, Salvo, Prado and Paz	Deposition at the infection sites	ND	10	1	G	Reduction in pathogen populations and progression of disease symptoms through therapeutic and prophylactic treatment with a phage cocktail.	[94]
Leek	*Pseudomonas syringae* pv. *porri*	Leek rust	KIL1,KIL3,KIL3b, KIL2,KIL4 and KIL5	Injection in leaves	ND	100	1	I	Individually, the phages reduced leaf lesions without complete disease protection.	[95]
				Immersion or spraying	Tween 20 (0.01%)	10	4 and 8	F	Phage cocktail treatment of seeds sown in a field previously inoculated with the pathogen did not result in significant protection. A reduction in symptomatic plants by 30% with weekly foliar spraying application of cocktail was observed.	
Kiwi	*Pseudomonas syringae* pv. *actinidiae*	Bacterial canker	CHF1, CHF7, CHF19, CHF21	Spraying	SM Buffer	10	1 or 2	G	Nearly halved the severity of disease on leaves in 2-year-old plants.	[96]
Mushroom *Pleurotus ostreatus*	*Pseudomonas tolaasii*	Brown spot	ϕPto-bp6g	Spraying	Water	0.01	1	I	Mushroom buds treated simultaneously with a pathogen and phage mixture exhibited the same growth as the pathogen-free control group, showing normal fruiting body development.	[97]
Geranium	*Xanthomonas campestris* pv. *pelargonii*	Bacterial blight	16 phages cocktail	Foliar spraying	ND	5 × 10^8^ PFU/mL	10 to 35	G	Reduction in disease plants from 53% to 19% with daily application. No protection with twice-weekly treatment. Similar protection with seedling protection.	[98]
Cherry tree	*Pseudomonas syringae* pv. *syringae*, and *morsprunorum*	Bacterial canker	MR6, MR8, MR15, MR16	Solution sprayed or applied on wounds or inoculated shoots	SM Buffer	0.01	1	C	Reduction in bacterial populations on leaves or in injured branches with single phages or in a cocktail.	[99]
Lemon tree	*Xanthomonas citri* subsp *citri*	Asian canker	6 phages originating from morphologically distinct lysis plaques	Spraying	Skimmed milk and saccharose (7.5 g/L; 2.5 g/L)	5 × 10^3^ PFU/mL	2	G	Reduction in disease symptoms by more than 50%with a phage cocktail application on 2-year-old lemon trees. Increased reduction using a plant defense activator.	[100]
					Water ± Actigard 500 WG/Skimmed milk and saccharose	5 × 10^3^ PFU/mL	2	F	Protection with cocktail and Actigard, and stronger protection with formulated cocktail.	
Grapefruit/orange tree	*Xanthomonas axonopodis* pv *citri and citrumelo*	Citrus canker and citrus bacterial spot	CP2, ccΦ7, ccΦ13, ΦXac2005-1	Spraying	Water ± 0.75% powdered milk	5 to 500	1	G	Reduction in disease severity without skimmed milk, none with formulated cocktail.	[101]
			ΦXaacA1	Spraying	Water + 0.75% powdered milk	10^6^ PFU/mL	24	F	Reduction in disease progression on moderately susceptible Valencia oranges, and no protection on highly susceptible grapefruit trees.	
Apple/pear tree	* Erwinia amylovora *	Bacterial fire blight	φEa1337-26	Spraying onto detached flowers	Phosphate buffer ± multiplying bacterium	100	1	G	Reduction in the infection by 60% with a non-pathogenic multiplying bacterium: *Pantoea agglomerans*.	[102]
			φEa2345-6, φEa2345-19 and φEa21-4	Same as above	Same as above	100		L	Reduction in infection by 88% to 97% with cocktail treatment	

ND: Not determined; ^(1)^: Phage-to-bacteria ratio; otherwise, use the applied phage concentration; ^(2)^: L: Laboratory, I: Incubator, C: Climatic chamber G: Greenhouse, F: field.

## Data Availability

No new data were created or analyzed in this study.
